# Causal effect between gut microbiota and metabolic syndrome in European population: a bidirectional mendelian randomization study

**DOI:** 10.1186/s13578-024-01232-6

**Published:** 2024-05-28

**Authors:** Jiawu Yan, Zhongyuan Wang, Guojian Bao, Cailin Xue, Wenxuan Zheng, Rao Fu, Minglu Zhang, Jialu Ding, Fei Yang, Beicheng Sun

**Affiliations:** 1 Department of Hepatobiliary Surgery, Innovative Institute of Tumor Immunity and Medicine (ITIM), The First Affiliated Hospital of Anhui Medical University, Hefei, Anhui 230022 China; 2 Anhui Province Key Laboratory of Tumor Immune Microenvironment and Immunotherapy, Hefei, China; 3 Nanjing Drum Tower Hospital, Affiliated Hospital of Medical School, Nanjing University, Nanjing, Jiangsu 210008 China; 4 Department of General Surgery, Jinling Hospital, Medical School of Nanjing University, Nanjing, China; 5 Division of Gastric Surgery, Department of General Surgery, the Affiliated Hospital of Medical School, Nanjing Drum Tower Hospital, Nanjing University, Nanjing, 210008 China; 6 Nanjing Drum Tower Hospital Clinical College of Nanjing University of Chinese Medicine, Nanjing, China

**Keywords:** Metabolic syndrome, Gut microbiota, Mendelian randomization study

## Abstract

**Background:**

Observational studies have reported that gut microbiota composition is associated with metabolic syndrome. However, the causal effect of gut microbiota on metabolic syndrome has yet to be confirmed.

**Methods:**

We performed a bidirectional Mendelian randomization study to investigate the causal effect between gut microbiota and metabolic syndrome in European population. Summary statistics of gut microbiota were from the largest available genome-wide association study meta-analysis (*n* = 13,266) conducted by the MiBioGen consortium. The summary statistics of outcome were obtained from the most comprehensive genome-wide association studies of metabolic syndrome (*n* = 291,107). The inverse-variance weighted method was applied as the primary method, and the robustness of the results was assessed by a series of sensitivity analyses.

**Results:**

In the primary causal estimates, *Actinobacteria* (OR = 0.935, 95% CI = 0.878–0.996, *P* = 0.037), *Bifidobacteriales* (OR = 0.928, 95% CI = 0.868–0.992, *P* = 0.028), *Bifidobacteriaceae* (OR = 0.928, 95% CI = 0.868–0.992, *P* = 0.028), *Desulfovibrio* (OR = 0.920, 95% CI = 0.869–0.975, *P* = 0.005), and *RuminococcaceaeUCG010* (OR = 0.882, 95% CI = 0.803–0.969, *P* = 0.009) may be associated with a lower risk of metabolic syndrome, while *Lachnospiraceae* (OR = 1.130, 95% CI = 1.016–1.257, *P* = 0.025), *Veillonellaceae* (OR = 1.055, 95% CI = 1.004–1.108, *P* = 0.034) and *Olsenella* (OR = 1.046, 95% CI = 1.009–1.085, *P* = 0.015) may be linked to a higher risk for metabolic syndrome. Reverse MR analysis demonstrated that abundance of *RuminococcaceaeUCG010* (OR = 0.938, 95% CI = 0.886–0.994, *P* = 0.030) may be downregulated by metabolic syndrome. Sensitivity analyses indicated no heterogeneity or horizontal pleiotropy.

**Conclusions:**

Our Mendelian randomization study provided causal relationship between specific gut microbiota and metabolic syndrome, which might provide new insights into the potential pathogenic mechanisms of gut microbiota in metabolic syndrome and the assignment of effective therapeutic strategies.

**Supplementary Information:**

The online version contains supplementary material available at 10.1186/s13578-024-01232-6.

## Introduction

Metabolic syndrome (MetS) is diagnosed when a person has at least three of the following five criteria: abdominal obesity, hyperglycemia, hypertension, high triglycerides and low HDL cholesterol levels [1]. MetS is an important world health problem that increases the risk of coronary heart disease, stroke, type 2 diabetes and overall mortality, and it affects about a quarter of the global population [2, 3]. Therefore, it is necessary to identify its causative risk factors in order to develop preventive and therapeutic strategies.

In recent years, there has been increasing evidence that gut microbiota disruption is a risk factor for the development of MetS [4]. The gut microbiota can influence host metabolism through various factors, such as defective gut barrier function, bile acid metabolism, antibiotic use, and the pleiotropic effects of metabolites produced by microbes [5]. Differences in gut microbiota exist between individuals with or without MetS and contribute to the progression of MetS [6, 7]. In addition, researchers have been working to investigate the possible therapeutic effects of the gut microbiota on MetS [8, 9]. Currently, therapies targeting the gut microbiota for the treatment of MetS mainly include probiotics and prebiotics, fecal microbiota transplantation, metabolic surgery, and application of drugs that can influence the gut microbiota [4].

However, while a causal relationship between gut microbial characterization and MetS has been established, controversy persists regarding their association with specific bacterial species. For instance, studies have shown that the proliferation of *Desulfovibrio* negatively impacts the colonization of beneficial Clostridia. Clostridia function to moderate the expression of CD36 and lipid absorption. Consequently, *Desulfovibrio* may contribute to obesity and MetS [10]. On the contrary, such a clear-cut relationship between *Desulfovibrio* and MetS is not always established. Actually, some literature reports an enrichment of *Desulfovibrio* in type 2 diabetes (T2D), while other studies suggest a negative correlation between *Desulfovibrio* and insulin resistance [11, 12]. Additionally, both an increase and decrease in *Desulfovibrio* levels may be associated with dietary interventions aimed at mitigating obesity or MetS [13–15]. Likewise, while Ciubotaru et al. initially reported higher levels of *Veillonellaceae* in prediabetes compared to normoglycemia groups [16], Lippert et al. did not observe such an association [17]. Therefore, further research is warranted to elucidate the relationship between different gut microbiota and MetS.

Mendelian randomization (MR) is an analytical method that uses genetic variants strongly associated with exposure as instrumental variables for modifiable risk factors affecting population health [18, 19]. It can overcome the bias caused by unmeasured confounding in observational studies [20, 21]. Therefore, in this study, we attempted to investigate the possible causal relationship between gut microbiota and MetS by two-sample MR analysis.

## Materials and methods

### The assumptions and study design of MR

**Fig. 1:**
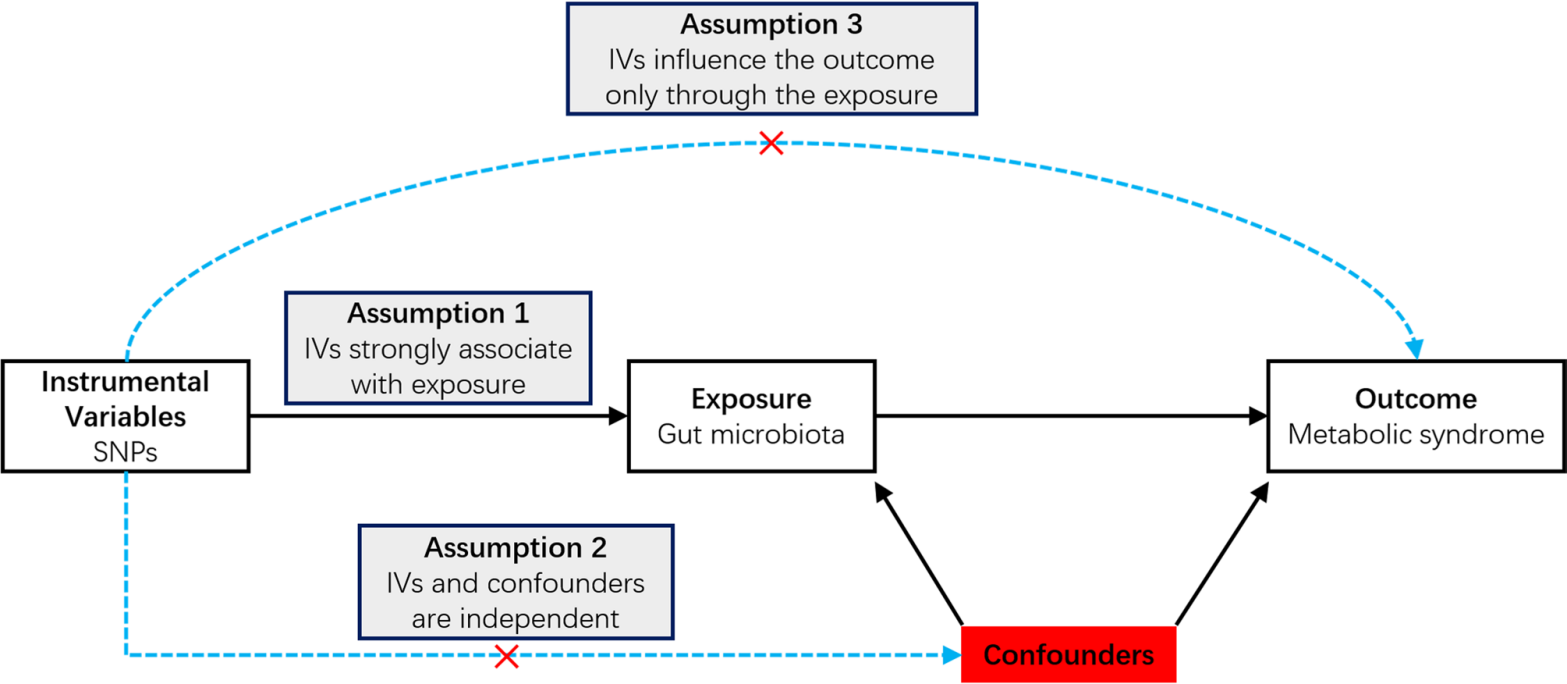
An overview of the study design. SNP: single nucleotide polymorphisms; IVs: instrumental variables

In this study, MR analysis was employed to investigate the potential causal link between the gut microbiota and MetS. We obtained summary-level data for the gut microbiota and MetS from the Genome-Wide Association Study (GWAS). Figure 1 shows the flowchart of the MR study.

To effectively demonstrate a causal effect, MR relies on three key assumptions: (1) Relevance assumption: the selected genetic Instrumental variables (IVs) were highly correlated with the exposure of interest (gut microbiota taxa); (2) Independence assumption: the included IVs were independent of confounders of the exposure-outcome association; (3) Exclusion restriction assumption: there was no horizontal pleiotropy, i.e., that the IVs affected the outcome only through their effect on gut microbiota taxa. Of note, we reported study results in strict accordance with the MRSTROBE guidelines. To minimize racial mismatch, we limited our analysis to participants of European descent.

### Data sources

We obtained the genetic variants for the gut microbiota from the largest genome-wide meta-analysis of gut microbiota composition published to date by the MiBioGen consortium [22]. The study included 18,340 individuals from 24 cohorts, most of whom were of European ancestry (*n* = 13,266), and targeted variable regions of the 16 S rRNA gene to profile the microbial composition. Classification was carried out using a direct taxonomic binning. Finally, a total of 211 taxa (9 phyla, 16 classes, 20 orders, 35 families and 131 genera) were included.

Summary-level data for MetS came from the most comprehensive GWAS in UK Biobank [23], comprising 291,107 individuals (59,677 cases and 231,430 controls) with no missing data on outcome, genotype, and covariate data. The diagnosis of MetS is based on uniform NCEP criteria that define five components of syndromic and epidemic MetS. Three of the following five criteria must be fulfilled: waist circumference > 102 cm in men and > 88 cm in women, serum glucose ≥ 6.1 mmol/L or antidiabetic treatment, Blood pressure ≥ 130/85 mmHg or antihypertensive treatment, serum triglycerides ≥ 1.7 mmol/L, HDL-cholesterol < 1.0 mmol/L in men and < 1.3 mmol/L in women [23, 24].

### Instrumental selection

The selection criteria for IVs were as follows: (1) Single nucleotide polymorphisms (SNPs) associated with each genus at the locus-wide significance threshold (P <${1.0\times 10}^{-5}$) were considered as potential IVs; (2) To satisfy the MR assumptions, we performed linkage disequilibrium (LD) analyses (R2 <0.001, clumping distance = 10,000 kb) based on European-based 1,000 Genome Projects and excluded non-compliant SNPs. (3) To prevent the influence of alleles on the outcome of causality between gut microbiota taxa and MetS, the palindromic SNPs were excluded. The F-statistic were calculated to assess the strength of IVs using the formula $\text{F}=\frac{{R}^{2}\times \left(N-1-K\right)}{\left(1-{R}^{2}\right)\times K}$, where ${R}^{2}$ represents the proportion of variance in the exposure explained by the genetic variants, N represents sample size, and K represents the number of instruments [25]. It was considered that there was no significant weak instrumental bias when the corresponding F-statistic was >10. We discarded IVs for which corresponding SNPs could not be found in the outcome GWAS dataset, owing to their exceedingly small count.

### Statistical analyses

We performed MR analyses to investigate the causal relationships between microbiome features and MetS. For features containing only one IV, the Wald ratio test was used to estimate the association between the identified IV and MetS. For features containing multiple IVs, five commonly used MR methods were used: inverse-variance weighted (IVW) test, Mendelian randomization Pleiotropy RESidual Sum and Outlier (MR-PRESSO), MR-RAPS, weighted median, and weighted mode. The IVW method has been reported to be slightly stronger than other methods under certain conditions [26]; therefore, the results with more than one IV were mainly based on the IVW method, supplemented by the other four methods. The MR-RAPS analysis has a robust loss function and the consideration of overdispersion and were accounted for weak instruments [27]. Weighted median analysis is more robust to individual genetic variation with strongly outlying causal estimates than the IVW and the MR-Egger methods [28]. Weighted mode analysis provides consistent estimates even when more than 50% of the instruments were invalid [29].

To assess the heterogeneity among SNPs associated with each microbial taxon, we performed Cochran’s Q test, and if the P value was higher than 0.05 and there was no evidence of heterogeneity, the fixed-effects IVW method was considered as the primary method. If substantial heterogeneity existed (*P* < 0.05), a random-effects IVW approach was used [30, 31]. To determine the presence of potentially strong effect SNPs, we conducted sensitivity analyses using the “leave-one-out” method to verify the reliability and stability of the causal effect estimates [32]. The MR-Egger intercept test can be used to assess the directional pleiotropy of selected IVs, and an intercept term that differs from zero in this method (tested here using a p value < 0.05) was indicative of an overall directional pleiotropy [33]. The MR-PRESSO method uses global and SNP-specific observation of residual sum of squares to test for general outliers and horizontal pleiotropy, respectively [34]. Finally, the results of causal associations were presented as odds ratios (ORs) and 95% confidence intervals (95% CI). Due to the presence of multiple tests, we corrected the P values using the FDR method, and only those with FDR < 0.05 were considered to have relatively convincing causal relationship, whereas results with FDR > 0.05 and *P* < 0.05 were considered to have a nominally significant causal relationship [35].

All statistical analyses were performed using R version 4.3.1 (R Foundation for Statistical Computing, Vienna, Austria). MR analyses were performed using the TwosampleMR (version 0.5.6), MR-PRESSO (version: 1.0), and mr.raps packages (version: 0.2).

## Results

### Selection of IVs

Based on the principles of IVs-selection, and after excluding 12 unknown genera, a total of 199 bacterial taxa (including 9 phyla, 16 classes, 20 orders, 35 families and 119 genera) containing 2223 SNPs ($\text{P}<1\times {10}^{-5}$) were finally identified as IVs in the MR analysis, and the details of all SNPs are detailed in Table S1. In the reverse MR analysis, we identified 71 SNPs of MetS as IVs (Table S5).

### Causal effects of Gut Microbiota on MetS

**Fig. 2:**
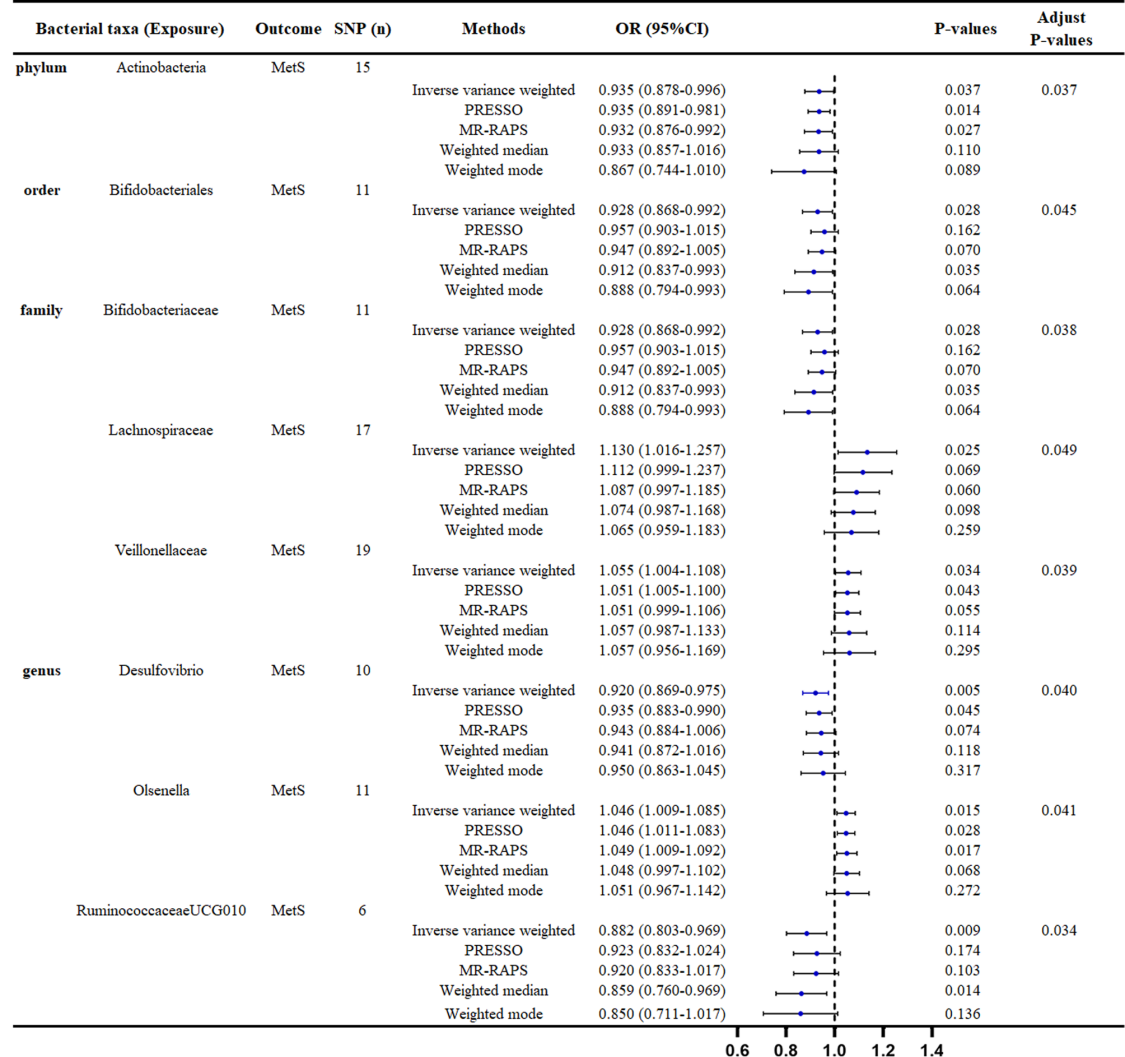
Positive MR results of causal links between gut microbiota on MetS. SNP: Single-nucleotide polymorphism; OR: Odds ratios; CI: Confidence interval; MetS: Metabolic syndrome; MR: Mendelian randomization; MR-PRESSO: Mendelian randomization Pleiotropy RESidual Sum and Outlier; MR-RAPS, Mendelian randomization-robust adjusted profile score

IVW was chosen as the primary method for MR analysis because of its higher statistical efficacy. As our result demonstrated, *phylum_Actinobacteria* (OR = 0.935, 95% CI = 0.878–0.996, *P* = 0.037), *order_Bifidobacteriales* (OR = 0.928, 95% CI = 0.868–0.992, *P* = 0.028), *family_Bifidobacteriaceae* (OR = 0.928, 95% CI = 0.868–0.992, *P* = 0.028), *genus_Desulfovibrio* (OR = 0.920, 95% CI = 0.869–0.975, *P* = 0.005), and *genus_RuminococcaceaeUCG010* (OR = 0.882, 95% CI = 0.803–0.969, *P* = 0.009) were protective factors for MetS. In contrast, *family_Lachnospiraceae* (OR = 1.130, 95% CI = 1.016–1.257, *P* = 0.025), *family_Veillonellaceae* (OR = 1.055, 95% CI = 1.004–1.108, *P* = 0.034) and *genus_Olsenella* (OR = 1.046, 95% CI = 1.009–1.085, *P* = 0.015) were predisposing factors for MetS (Fig. 2).

### Sensitivity analysis

**Fig. 3:**
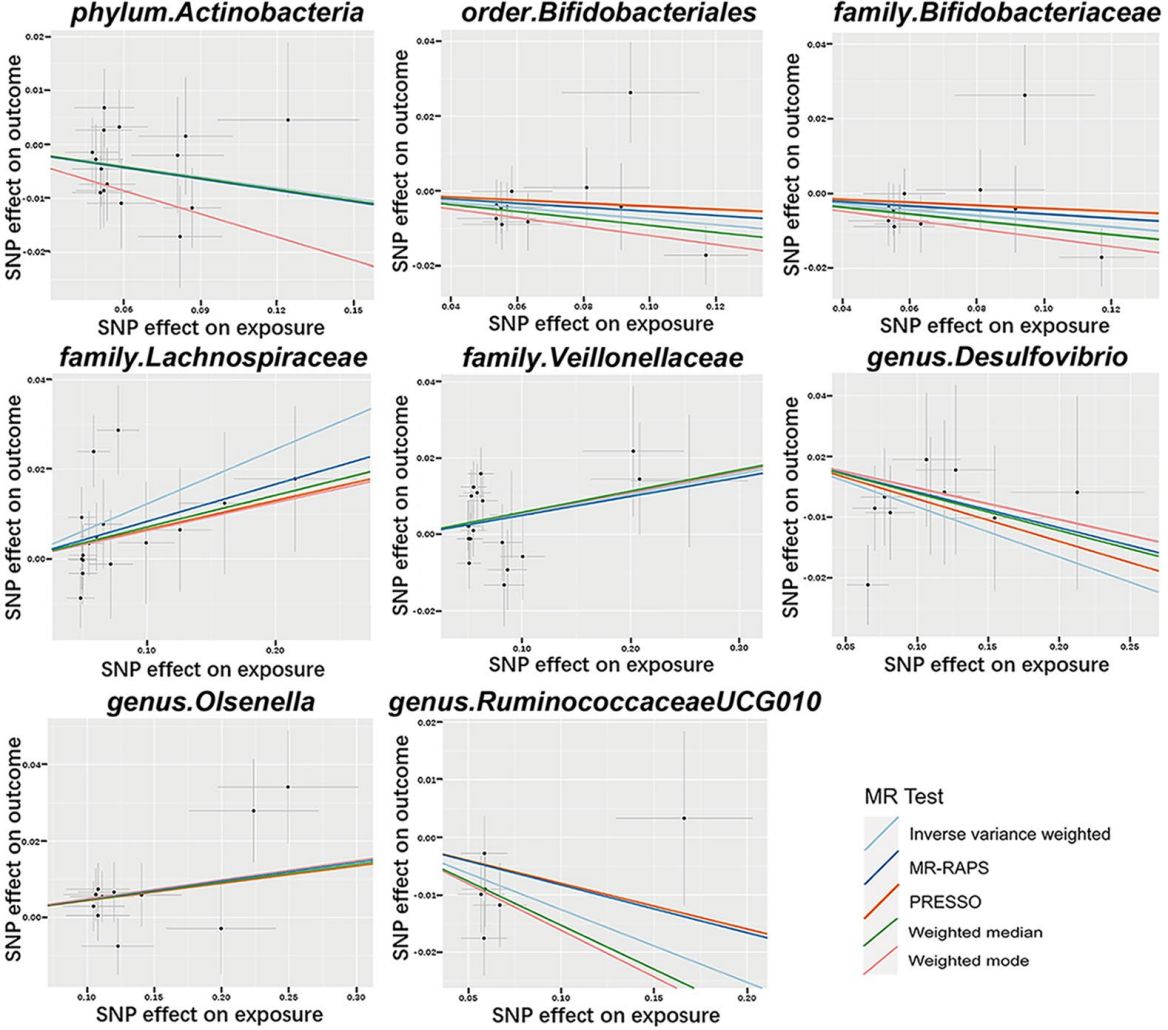
Scatter plots for MR analyses of the causal effect of gut microbiota on MetS. SNP: Single-nucleotide polymorphism; MetS: Metabolic syndrome; MR: Mendelian randomization; MR-PRESSO: Mendelian randomization Pleiotropy RESidual Sum and Outlier; MR-RAPS: Mendelian randomization-robust adjusted profile score

**Fig. 4:**
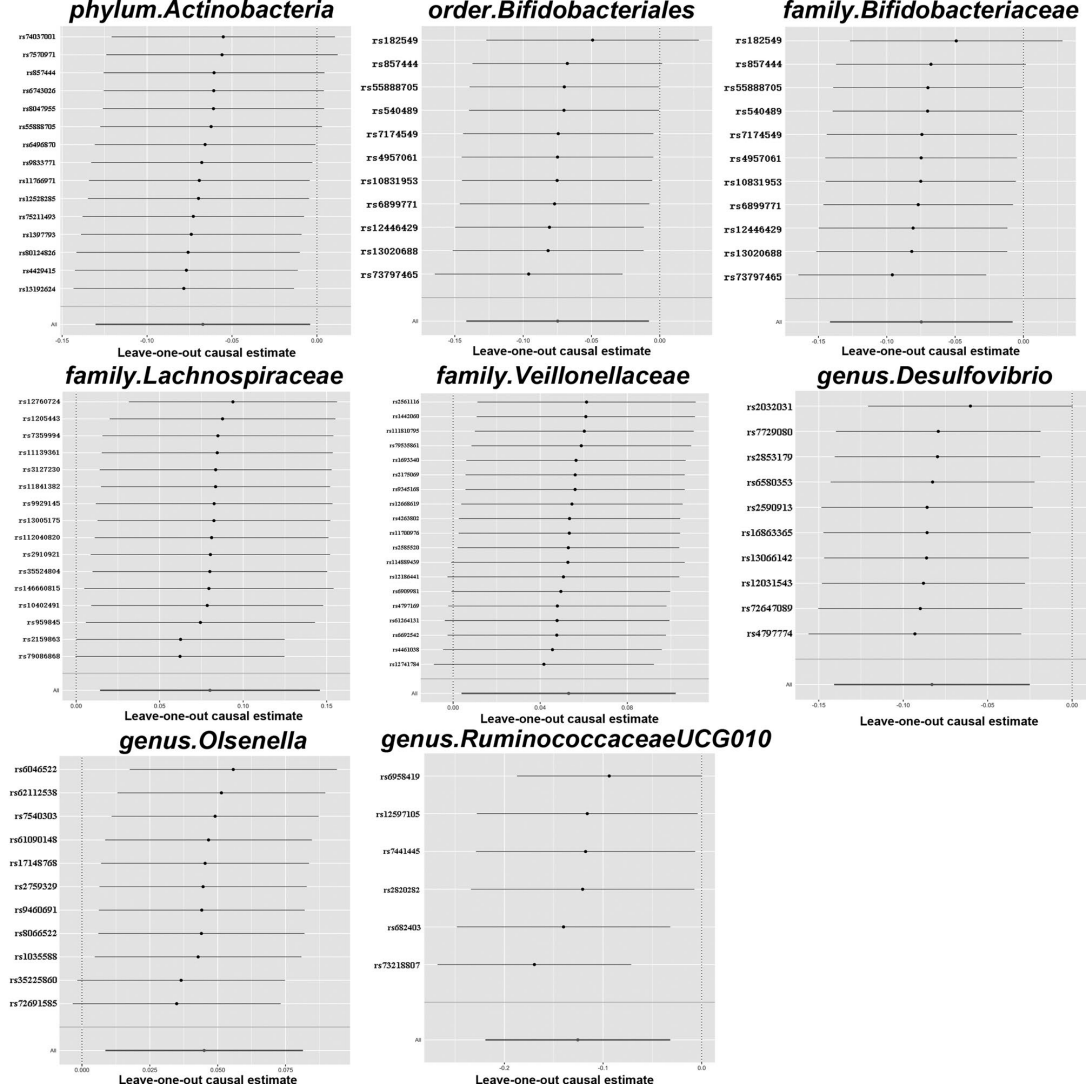
The leave-one-out plots for MR analyses of the causal effect of gut microbiota on MetS. MR: Mendelian randomization; MetS: Metabolic syndrome

In these causal effects mentioned above, the F-statistics for IVs were all >10 (Fig. 2, Table S1), indicating there was no weak instrument bias. Through Cochran’s Q tests, no heterogeneity (${P}_{h}$>0.05) were found, except for *family.Lachnospiraceae*, whose ${P}_{h}$<0.05 (Table S2). So we chose the random-effects IVW approach to reestimate the causal effect and the result was *family_Lachnospiraceae* (OR = 1.130, 95% CI = 1.016–1.257, *P* = 0.025). All P-value of MR–Egger interpret was > 0.05, showing the absence of horizontal pleiotropy (Table S3). The Scatter plots (Fig. 3) demonstrates the causal effect of gut microbiota on MetS. Further MR-PRESSO analysis did not reveal any significant outliers (Table S4). However, leave-one-out (Fig. 4) analysis showed that the change of significance due to the exclusion of one SNP was observed in several exposures, including *phylum_Actinobacteria*, *order_Bifidobacteriales*, *family_Bifidobacteriaceae*, *family_Veillonellaceae* and *genus_Olsenella*. To further test the robustness of our conclusion, we conducted further sensitivity analysis by specifying a more stringent instrumental variable selection scheme. The results were presented in the Supplementary Data.

### Reverse MR analysis

According to the results of reverse MR analysis, there was a suggestive association between MetS and *RuminococcaceaeUCG010* (OR = 0.938, 95% CI = 0.886–0.994, *P* = 0.030). No significant causal association was found between MetS and the other gut microbiota. The results of MR Egger regression intercepted item analysis and MR-PRESSO analysis also did not find significant horizontal pleiotropy (Table S5).

## Discussion

In this present study, we analyzed the causal effect between gut microbiome and MetS using a bidirectional MR method based on publicly available GWAS database. Our findings revealed that *phylum_Actinobacteria*, *order_Bifidobacteriales*, *family_Bifidobacteriaceae*, *genus_Desulfovibrio*, and *genus_RuminococcaceaeUCG010* were protective factors, while *family_Lachnospiraceae* and *family_Veillonellaceae* were associated with increased risk for MetS.

Observational epidemiological studies encounter various biases, such as confounding and reverse causation, which constrain their capacity to firmly establish causal links [36]. Compared to observational studies, the avoidance of confounding is clearly a key advantage of MR [37]. In MR, the underlying concept suggests that if SNPs result in phenotypic variations mirroring the biological impacts of modifiable environmental exposures, which subsequently affect disease risk, then these SNPs themselves should correlate with disease risk proportionally to their influence on the phenotype. Therefore, common polymorphisms with well-defined biological functions can serve to examine the impact of suspected exposures on disease risk. An important consideration is that the distribution of such polymorphisms is largely independent of confounding variables—such as socioeconomic or behavioral factors—that have been identified previously as distorting interpretations of results from observational epidemiological studies [20]. These make MR an important method for causal inference.

The human gut microbiome is a complex ecosystem that can influence normal physiology of the body by affecting metabolism and immune system [38]. Recently, several observational studies have reported the association between gut microbiota and MetS [4, 5]. For example, Lim, M.Y., et al. found that the *Bifidobacteriaceae* were enriched in healthy populations compared to MetS patients. Besides, the phylum *Actinobacteria*, to which *Bifidobacteriaceae* belongs, had a high heritability (45.7%) [39]. *Actinobacteria*, especially the *Bifidobacteria*, exhibited a protective role in HFD induced diabetes [40, 41]. As a probiotic, *Bifidobacterium* has also been widely reported to have a protective effect against metabolic diseases [42]. Our results are consistent with these findings, showing that the phylum *Actinobacteria*, as well as its constituent *Bifidobacteriaceae*, act as protective factors for MetS, exhibiting a negative correlation with MetS. Mechanically, *Bifidobacteriaceae* are involved in the metabolism of short-chain fatty acids (SCFA) [43, 44]. SCFAs, mostly acetic acid, propionic acid, and butyric acid, are the major end-products of metabolism by the intestinal microbiota in the human body [45]. SCFA can regulate blood pressure by a variety of mechanisms, but mainly through the activation of transmembrane G protein-coupled receptors (GPCR), including CPR41, CPR43, and olfactory receptor 78 (Olfr78) [46].

In addition, previous studies have shown reduced abundance of *Ruminococcaceae* in the MetS model [47], and consistent with this, our results of reverse MR analysis also exhibited a negative association between MetS and *Ruminococcaceae* abundance. Furthermore, reduced *Ruminococcaceae* abundance is inversely associated with clinical indicators of metabolic diseases, and reduced abundance of *Ruminococcaceae* was also associated with the development of obesity [17, 48]. From a mechanistic standpoint, *Ruminococcaceae* are known to be significant consumers of plant polysaccharides, leading to the production of butyrate [49]. *Ruminococcaceae* exerts a regulatory influence on lipid metabolism, diminishes inflammation, fortifies intestinal barrier integrity, curbs weight gain, and enhances insulin sensitivity in mice, effectively impeding the progression of obesity [50]. Thus prevalence of *Ruminococcaceae* has been associated with reduced endotoxemia and inversely correlated with metabolic disorders [51]. In our findings, we observed that *Ruminococcaceae* may exert a protective role against MetS. This is corroborated by the aforementioned studies. Additionally, our reverse MR analysis further validated that MetS could lead to downregulation of *Ruminococcaceae*. Therefore, both published results and our findings suggest that *Ruminococcaceae* may be involved in MetS in such a way that the occurrence of MetS leads to a reduction in *Ruminococcaceae*, thereby attempting to suppress or alleviate the further progression of MetS. In summary, these findings may suggest that *Ruminococcaceae* could play a more significant role in the prevention or treatment of MetS than currently demonstrated, and further research may be needed to confirm this.

We have also identified certain bacterial taxa that show a positive correlation with an elevated risk of MetS. Specifically, we have identified *Lachnospiraceae* as a risk factor associated with MetS. Consistent with our result, an earlier study demonstrated elevated levels of *Lachnospiraceae* in patient groups with obesity [52], impaired glucose metabolism and/or MetS [17, 53]. Mechanistically, in both human and animal studies, higher levels of *Lachnospiraceae* have been linked to metabolic disorders, potentially attributed to the production of short-chain fatty acids (SCFAs) other than butyrate [54, 55]. Emerging evidence suggests that specific SCFAs (e.g. acetate and propionate) have pathological effects in various diseases, including obesity [56, 57]. Moreover, a reduction in the abundance of *Lachnospiraceae_NK4A136_group* was linked to improvements in obesity [14].

Another bacterial taxa we identified as positively correlated with MetS is *Olsenella*. The *Olsenella* is important in immunotherapy as it significantly enhances the efficacy of immune checkpoint inhibitors [58]. However, the association between *Olsenella* and metabolic diseases still requires further investigation. Therefore, our results provide a reference for further exploring the relationship between *Olsenella* and MetS. Several studies have indicated a significant association between *Olsenella* and dysbiosis as well as inflammation [59–61]. Dysbiosis and inflammation in the gut can compromise the integrity of the intestinal barrier, rendering the intestinal epithelium more vulnerable to microbial lipopolysaccharides, trimethylamine, and other metabolites that enter the bloodstream, contributing to pathologies related to MetS [5, 62]. This provides a potential explanation for the causal relationship between *Olsenella* and MetS, as revealed by our findings. However, further research is needed to elucidate the relationship between *Olsenella* and MetS, as well as the underlying mechanisms.

It is noteworthy that for two bacterial taxa, *Desulfovibrio* and *Veillonellaceae*, whose relationship with MetS is controversial, our results provide valuable reference for establishing their correlation. As previously mentioned, studies have presented conflicting findings, with some indicating an increase in *Desulfovibrio* abundance in T2D and others suggesting a potential inverse relationship between *Desulfovibrio* levels and insulin resistance [10, 11]. Similarly, the abundance of *Veillonellaceae* has been reported to be either downregulated or upregulated in individuals with metabolic disorders [15, 16]. The contradictory associations between gut microbiota taxa and MetS could stem from potential biases arising from unmeasured or unknown confounding factors. To address these limitations, our MR analysis utilized large-scale GWAS summary statistics and genetic instruments unaffected by confounding factors or reverse causation. Through this robust approach, we confirmed the negative causal association between *Desulfovibrio* and MetS, as well as the positive causal relationship between *Veillonellaceae* and MetS. In summary, our findings not only provide valuable insights but also lay the groundwork for further investigation into the role of *Desulfovibrio* and *Veillonellaceae* in metabolic disorders.

Our study utilized MR to estimate the causal relationship between gut microbiota and MetS, and identified several kinds of bacterial taxa associated with MetS. Our findings provide a reference for further research on the correlation between gut microbiota and MetS, as well as the development of bacterial-related therapies for MetS. Despite the valuable insights gained from this study, there are some limitations to our research. First, the Mibiogen database, source of gut microbiota GWAS, is the largest multi-ethnic genome-wide meta-analysis of gut microbiota, but it includes not just individuals of European ancestry. This heterogeneity may influence the reliability and generalizability of our conclusions. Second, because the lowest taxonomic level in the exposure dataset was genus, this limited our ability to further explore the causal relationships at the species level. Third, although the summary statistics of gut microbiota that we selected are from the largest available genome-wide association study meta-analysis, due to database limitations, the number of patients per specific gut microbiota species in our study was relatively small. This resulted in a very small number of eligible IVs meeting the traditional GWAS significance threshold (*P* < 5 × 10e − 8), so we chose a relatively more comprehensive threshold (*P* < 1 × 10e − 5) to obtain more comprehensive results. And this adjustment may introduce some bias into the conclusions. Fourth, although the main reason to approach MR is to avoid the problem of residual confounding, MR studies cannot fully consider possible confounding factors [37]. It is difficult to ensure that each SNP satisfies the three IVs assumptions. Despite efforts, unknown confounders may still exist, leading to violations of these assumptions and potentially affecting the results of analyses [63]. As a result, potential confounding of the genetic variants and the outcome can occur in MR studies. This means that caution should be exercised when analyzing and utilizing some results of Mendelian randomization [64]. Finally, it should be recognized that the conclusions reached in this study have not been externally validated in a clinical setting. This is a limitation that should be recognized.

## Conclusions

In conclusion, our two-sample MR study suggested a potential presence of a causal effect between gut microbiota and MetS. Specifically, our findings revealed that *Actinobacteria*, *Bifidobacteriales*, *Desulfovibrio* and *RuminococcaceaeUCG010* were associated with downregulated risk of MetS, while *Lachnospiraceae* and *Veillonellaceae* were associated with increased risk of MetS. In addition, reverse MR analysis supported a negative causal association between MetS and *RuminococcaceaeUCG010*. However, more studies are needed to support the findings of our current study.

### Electronic supplementary material

Below is the link to the electronic supplementary material.

**Supplementary Material 1:** Table S1

**Supplementary Material 2:** Table S2

**Supplementary Material 3:** Table S3

**Supplementary Material 4:** Table S4

**Supplementary Material 5:** Table S5

**Supplementary Material 6:** Supplementary data

**Supplementary Material 7:** STROBE checklist

## Data Availability

The datasets analyzed during the current study are available in the MiBioGen repository (https://mibiogen.gcc.rug.nl/) and the Met GWAS are available in https://www.ebi.ac.uk/gwas/efotraits/EFO_0000195.

## Abbreviations

CI: Confidence interval
IVW: Inverse variance weighted
MetS: Metabolic syndrome
MR: Mendelian randomization
OR: Odds ratios
SNPs: Single-nucleotide polymorphisms
GWAS: genome-wide relationship studies
IVs: Instrumental variables
MR-PRESSO: Mendelian randomization Pleiotropy RESidual Sum and Outlier
FDR: False discovery rate
