# Supplementary material for: Causal effect between gut microbiota and metabolic syndrome in European population: a bidirectional mendelian randomization study
Source: Cell Biosci. 2024 May 28;14:67. doi: 10.1186/s13578-024-01232-6 (PMC11134679; doi:10.1186/s13578-024-01232-6)

Supplementary table 1. Further sensitivity analysis performed on the exposures that exhibited significant changes upon excluding a single SNP. IVW: inverse variance–weighted; SNP: single nucleotide polymorphism; OR: odds ratio; CI: confidence intervals; MetS: metabolic syndrome.

Supplementary figure 1. Leave-one-out plots for MR analyses of the causal effect of *phylum.Actinobacteria* on MetS in Table 1. MR: Mendelian Randomization; MetS: metabolic syndrome.

**phylum.Actinobacteria**


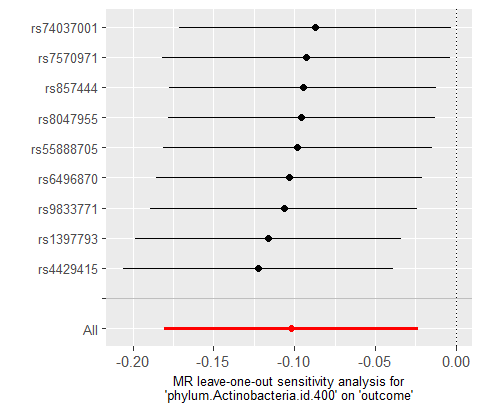

Supplement: Supplementary file 6 — Supplementary Material 6: Supplementary data [file 13578_2024_1232_MOESM6_ESM.docx]
