# Supplementary material for: Causal effect between gut microbiota and metabolic syndrome in European population: a bidirectional mendelian randomization study
Source: Cell Biosci. 2024 May 28;14:67. doi: 10.1186/s13578-024-01232-6 (PMC11134679; doi:10.1186/s13578-024-01232-6)
Supplement: Supplementary file 7 — Supplementary Material 7: STROBE checklist [file 13578_2024_1232_MOESM7_ESM.docx]

**STROBE-MR checklist of recommended items to address in reports of Mendelian randomization studies**^1^ ^2^

| **Item No.** | **Section** | **Checklist item** | **Page No.** | **Relevant text from manuscript** |
| --- | --- | --- | --- | --- |
| 1 | **TITLE and ABSTRACT** | Indicate Mendelian randomization (MR) as the study’s design in the title and/or the abstract if that is a main purpose of the study | 1,2 | Causal effect between gut microbiota and metabolic syndrome in European populations: a bidirectional Mendelian randomization study  ABSTRACT |
|  | **INTRODUCTION** |  | 2 | INTRODUCTION |
| 2 | **Background** | Explain the scientific background and rationale for the reported study. What is the exposure? Is a potential causal relationship between exposure and outcome plausible? Justify why MR is a helpful method to address the study question | 1 | Background |
| 3 | **Objectives** | State specific objectives clearly, including pre-specified causal hypotheses (if any). State that MR is a method that, under specific assumptions, intends to estimate causal effects | 3 | Therefore, in this study, we attempted to investigate the possible causal relationship between gut microbiota and MetS by two-sample MR analysis. |
|  | **METHODS** |  |  |  |
| 4 | **Study design and data sources** | Present key elements of the study design early in the article. Consider including a table listing sources of data for all phases of the study. For each data source contributing to the analysis, describe the following: |  |  |
|  | a) | Setting: Describe the study design and the underlying population, if possible. Describe the setting, locations, and relevant dates, including periods of recruitment, exposure, follow-up, and data collection, when available. | 1 | We performed a bidirectional Mendelian randomization study to investigate the causal effect between gut microbiota and metabolic syndrome in European populations. |
|  | b) | Participants: Give the eligibility criteria, and the sources and methods of selection of participants. Report the sample size, and whether any power or sample size calculations were carried out prior to the main analysis | 3 | The study included 18,340 individuals from 24 cohorts, most of whom were of European ancestry (n = 13,266) |
|  | c) | Describe measurement, quality control and selection of genetic variants | 4 | Instrumental selection |
|  | d) | For each exposure, outcome, and other relevant variables, describe methods of assessment and diagnostic criteria for diseases | 3 | The diagnosis of MetS is based on uniform NCEP criteria |
|  | e) | Provide details of ethics committee approval and participant informed consent, if relevant | 10 | Ethics approval and consent to participate |
| 5 | **Assumptions** | Explicitly state the three core IV assumptions for the main analysis (relevance, independence and exclusion restriction) as well assumptions for any additional or sensitivity analysis | 3 | The assumptions and study design of MR |
| 6 | **Statistical methods: main analysis** | Describe statistical methods and statistics used |  |  |
|  | a) | Describe how quantitative variables were handled in the analyses (i.e., scale, units, model) |  |  |
|  | b) | Describe how genetic variants were handled in the analyses and, if applicable, how their weights were selected | 4 | Instrumental selection |
|  | c) | Describe the MR estimator (e.g. two-stage least squares, Wald ratio) and related statistics. Detail the included covariates and, in case of two-sample MR, whether the same covariate set was used for adjustment in the two samples |  |  |
|  | d) | Explain how missing data were addressed | 4 | We discarded IVs for which corresponding SNPs could not be found in the outcome GWAS dataset, owing to their exceedingly small count. |
|  | e) | If applicable, indicate how multiple testing was addressed |  |  |
| 7 | **Assessment of assumptions** | Describe any methods or prior knowledge used to assess the assumptions or justify their validity | 4 | The selection criteria for IVs were as follows: |
| 8 | **Sensitivity analyses and additional analyses** | Describe any sensitivity analyses or additional analyses performed (e.g. comparison of effect estimates from different approaches, independent replication, bias analytic techniques, validation of instruments, simulations) | 4 | Statistical analyses |
| 9 | **Software and pre-registration** |  |  |  |
|  | a) | Name statistical software and package(s), including version and settings used | 5 | All statistical analyses were performed using R version 4.3.1 (R Foundation for Statistical Computing, Vienna, Austria). MR analyses were performed using the TwosampleMR (version 0.5.6), MR-PRESSO (version: 1.0), and mr.raps packages (version: 0.2). |
|  | b) | State whether the study protocol and details were pre-registered (as well as when and where) |  |  |
|  | **RESULTS** |  |  |  |
| 10 | **Descriptive data** |  |  |  |
|  | a) | Report the numbers of individuals at each stage of included studies and reasons for exclusion. Consider use of a flow diagram | 5 | Selection of IVs |
|  | b) | Report summary statistics for phenotypic exposure(s), outcome(s), and other relevant variables (e.g. means, SDs, proportions) |  |  |
|  | c) | If the data sources include meta-analyses of previous studies, provide the assessments of heterogeneity across these studies |  |  |
|  | d) | For two-sample MR:  i.  Provide justification of the similarity of the genetic variant-exposure associations between the exposure and outcome samples  ii.  Provide information on the number of individuals who overlap between the exposure and outcome studies | 3 | Data sources |
| 11 | **Main results** |  |  |  |
|  | a) | Report the associations between genetic variant and exposure, and between genetic variant and outcome, preferably on an interpretable scale | 5 | Based on the principles of IVs-selection, and after excluding 12 unknown genera, a total of 199 bacterial taxa (including 9 phyla, 16 classes, 20 orders, 35 families and 119 genera) containing 2223 SNPs (P<1×〖10〗^(-5)) |
|  | b) | Report MR estimates of the relationship between exposure and outcome, and the measures of uncertainty from the MR analysis, on an interpretable scale, such as odds ratio or relative risk per SD difference | 5 | Causal Effects of Gut Microbiota on MetS |
|  | c) | If relevant, consider translating estimates of relative risk into absolute risk for a meaningful time period |  |  |
|  | d) | Consider plots to visualize results (e.g. forest plot, scatterplot of associations between genetic variants and outcome versus between genetic variants and exposure) | 13 | Figure3 |
| 12 | **Assessment of assumptions** |  |  |  |
|  | a) | Report the assessment of the validity of the assumptions | 4 | The selection criteria for IVs were as follows: (1) Single nucleotide polymorphisms (SNPs) associated with each genus at the locus-wide significance threshold (P <〖1.0×10〗^(-5)) were considered as potential IVs; (2) To satisfy the MR assumptions, we performed linkage disequilibrium (LD) analyses (R2 <0.001, clumping distance = 10,000kb) based on European-based 1,000 Genome Projects and excluded non-compliant SNPs. (3) To prevent the influence of alleles on the outcome of causality between gut microbiota taxa and MetS, the palindromic SNPs were excluded. |
|  | b) | Report any additional statistics (e.g., assessments of heterogeneity across genetic variants, such as *I^2^*, Q statistic or E-value) | 5 | Through Cochran’s Q tests, no heterogeneity (P_h>0.05) were found |
| 13 | **Sensitivity analyses and additional analyses** |  |  |  |
|  | a) | Report any sensitivity analyses to assess the robustness of the main results to violations of the assumptions | 6 | leave-one-out |
|  | b) | Report results from other sensitivity analyses or additional analyses |  |  |
|  | c) | Report any assessment of direction of causal relationship (e.g., bidirectional MR) | 6 | Reverse MR analysis |
|  | d) | When relevant, report and compare with estimates from non-MR analyses | 6 | DISCUSSION |
|  | e) | Consider additional plots to visualize results (e.g., leave-one-out analyses) | 14 | Figure4 |
|  | **DISCUSSION** |  |  |  |
| 14 | **Key results** | Summarize key results with reference to study objectives | 6 | Our findings revealed that phylum_Actinobacteria, order_Bifidobacteriales, family_Bifidobacteriaceae, genus_Desulfovibrio, and |
| 15 | **Limitations** | Discuss limitations of the study, taking into account the validity of the IV assumptions, other sources of potential bias, and imprecision. Discuss both direction and magnitude of any potential bias and any efforts to address them | 8 | there are some limitations to our research |
| 16 | **Interpretation** |  |  |  |
|  | a) | Meaning: Give a cautious overall interpretation of results in the context of their limitations and in comparison with other studies | 9 | However, more studies are needed to support the findings of our current study. |
|  | b) | Mechanism: Discuss underlying biological mechanisms that could drive a potential causal relationship between the investigated exposure and the outcome, and whether the gene-environment equivalence assumption is reasonable. Use causal language carefully, clarifying that IV estimates may provide causal effects only under certain assumptions | 7 | Mechanically, Bifidobacteriaceae are involved in the metabolism of short-chain fatty acids (SCFA) [45, 46]. SCFAs, mostly acetic acid, propionic acid, and butyric acid, are the major end-products of metabolism by the intestinal microbiota in the human body [47]. |
|  | c) | Clinical relevance: Discuss whether the results have clinical or public policy relevance, and to what extent they inform effect sizes of possible interventions | 9 | Our findings provide a reference for further research on the correlation between gut microbiota and MetS, as well as the development of bacterial-related therapies for MetS. |
| 17 | **Generalizability** | Discuss the generalizability of the study results (a) to other populations, (b) across other exposure periods/timings, and (c) across other levels of exposure |  |  |
|  | **OTHER INFORMATION** |  |  |  |
| 18 | **Funding** | Describe sources of funding and the role of funders in the present study and, if applicable, sources of funding for the databases and original study or studies on which the present study is based | 10 | Funding |
| 19 | **Data and data sharing** | Provide the data used to perform all analyses or report where and how the data can be accessed, and reference these sources in the article. Provide the statistical code needed to reproduce the results in the article, or report whether the code is publicly accessible and if so, where | 10 | Data Availability |
| 20 | **Conflicts of Interest** | All authors should declare all potential conflicts of interest | 10 | Competing interests |

This checklist is copyrighted by the Equator Network under the Creative Commons Attribution 3.0 Unported (CC BY 3.0) license.

1. Skrivankova VW, Richmond RC, Woolf BAR, Yarmolinsky J, Davies NM, Swanson SA, et al. Strengthening the Reporting of Observational Studies in Epidemiology using Mendelian Randomization (STROBE-MR) Statement. JAMA. 2021;under review.

2. Skrivankova VW, Richmond RC, Woolf BAR, Davies NM, Swanson SA, VanderWeele TJ, et al. Strengthening the Reporting of Observational Studies in Epidemiology using Mendelian Randomisation (STROBE-MR): Explanation and Elaboration. BMJ. 2021;375:n2233.
